# DNA damage response inhibitors in pancreatic cancer: progress and challenges

**DOI:** 10.3389/fonc.2026.1838580

**Published:** 2026-06-15

**Authors:** Yeyao Wu, Wei Li, Mengyun Wu

**Affiliations:** Radiology Laboratory, Department of Occupational Health and Radiological Health, Chongqing Center for Disease Control and Prevention, Chongqing, China

**Keywords:** ATR inhibitors, combination therapy, DDR inhibitors, DNA damage response (DDR), homologous recombination deficiency (HRD), pancreatic cancer, pancreatic ductal adenocarcinoma, PARP inhibitors

## Abstract

Pancreatic cancer is one of the most lethal malignancies, with very limited treatment options beyond standard chemotherapy. The discovery of homologous recombination repair (HRR) deficiencies—such as BRCA1/2 and PALB2 mutations—has revealed a targetable vulnerability through the DNA damage response (DDR) pathway. This review systematically summarizes the application and current clinical landscape of DDR−targeted therapies in pancreatic cancer. We first discuss how DDR inhibitors have successfully translated from research into clinical practice as a targeted treatment option for pancreatic cancer patients. However, single−agent DDR inhibitors face major limitations, including a narrow beneficiary population, primary and acquired resistance, and dose−limiting toxicities. The central theme of this review is the paradigm shift from monotherapy to rational combination strategies. We therefore focus on emerging combination approaches, such as the PAD/PAD_tal_ triple regimen, dual−target inhibitors, sequential scheduling, combinations with immunotherapy, combinations with KRAS inhibitors, and novel targets (PRMT5, PARG, HuR). These strategies aim to overcome resistance, extend benefits beyond BRCA−mutant patients, and remodel the tumor immune microenvironment. Finally, we summarize current challenges—including limited accessibility, resistance mechanisms, and toxicities—and outline future directions, such as novel biomarkers, liquid biopsy for real−time resistance monitoring, and innovative adaptive trial designs. This review highlights the evolution of DDR−targeted therapy in pancreatic cancer toward personalized, combination−based precision medicine.

## 1 Introduction

Pancreatic cancer ranks among the most fatal cancers globally, with its incidence rate nearly equaling its mortality rate (1). Although diagnosis and treatment methods have advanced, the outlook for pancreatic cancer has improved little: the overall 5−year survival rate remains below 10%, and even in regions with advanced medical care, it hovers around 12%—still below 20% (2, 3). Patients typically receive a diagnosis at a late stage, and the cancer inherently resists chemotherapy. Therefore, we urgently need new treatment strategies grounded in a deeper understanding of tumor biology.

The high mortality of pancreatic cancer stems from three key reasons. First, the disease starts slowly, so it is hard to find early (4). The pancreas lies deep in the body, and early symptoms—such as upper abdominal discomfort or indigestion—are non−specific, mimicking common gastrointestinal disorders (5). Currently, there is a lack of efficient and cost-effective early screening methods in clinical practice. As a result, more than 80% of patients are already at a locally advanced or metastatic stage when they are found, which results in missing the best time for surgery to remove the tumor (6). Second, treatment options are limited, and the usual ones do not work well. Surgery remains the only potentially curative approach, but only 15%–20% of patients have resectable tumors at initial diagnosis (7, 8). Even among those who undergo surgery followed by adjuvant chemotherapy, the recurrence rate stays extremely high (9). For most patients with locally advanced or metastatic cancer, systemic chemotherapy is the mainstay, yet it offers limited benefit (10). Moreover, real−world data reveal an even grimmer picture: a 2024 nationwide French study reported a median survival of only 8.5 months for all pancreatic cancer patients, and even among patients without metastasis, the 1−year survival rate was only 68.8% (11). Third, pancreatic cancer grows and spreads in a very aggressive way. Pancreatic cancer cells readily invade nearby tissues and tend to infiltrate nerves and blood vessels early, which explains the disease’s early dissemination, short natural course, and rapid progression (12). The facts above show that making new treatments is urgent and hard. A retrospective analysis by Pishvaian et al. showed that precision medicine can affect survival in patients with pancreatic cancer, and that molecularly guided treatments targeting oncogenic drivers and the DNA damage response (DDR) pathway warrant further prospective evaluation (13). We therefore need a better understanding of tumor biology, particularly its genomic instability and reliance on DDR pathways, to identify targeted treatment strategies.

The DDR is a complex signaling network responsible for maintaining genomic integrity by coordinating DNA repair and cell cycle checkpoints to prevent the transmission of damage to daughter cells (14, 15). Upon exposure to endogenous or exogenous DNA damage, the DDR pathway starts a series of steps. First, sensor proteins such as ATM and ATR detect the damage, relay signals, arrest the cell cycle at checkpoints to allow time for repair, and then activate the appropriate repair process (16, 17). These repair pathways include homologous recombination repair (HRR), non-homologous end joining (NHEJ), base excision repair (BER), and mismatch repair (MMR), collectively working as a defense system to keep DNA stable (18–21).

Dysfunctional DDR pathways promote tumor formation (22). When DDR function is broken, cells cannot repair DNA damage the right way or on time, leading to genomic instability and increased mutations that drive tumorigenesis (23). However, this weak spot also creates a unique therapeutic opportunity for cancer treatment: if DDR pathways are not working, tumor cells may rely on backup repair pathways, so those backup pathways become targets for drugs (24). DDR-targeted inhibitors are made using the idea of “synthetic lethality.” they block the alternative pathways that cancer cells depend on for survival, thereby killing cancer cells in a targeted manner (25).

In recent years, DDR inhibitors have shown promise in many cancer types, particularly those with defects in key DNA repair genes. PARP inhibitors are the most successful example. They are now approved to treat breast, ovarian, pancreatic, and prostate cancers with BRCA mutations (26–29). Inhibitors targeting other DDR components—such as ATR, CHK1/2, WEE1, and DNA−PK—are under investigation in preclinical and early−Phase clinical studies. And studies have found that DDR pathways and the immune system are closely linked. DDR inhibitors activate the cGAS−STING pathway, promoting type I interferon production and T cell recruitment into tumors, which provides a rationale for combining DDR inhibitors with immunotherapy (30, 31). Despite substantial progress in DDR−targeted therapies, clinical application still faces obstacles, including drug resistance and a limited patient population that can benefit. To improve treatments and reach more patients, we need to understand how DDR pathways function at the molecular level and how we can leverage them for cancer therapy.

## 2 Key DDR pathway gene alterations in pancreatic cancer

**Figure 1:**
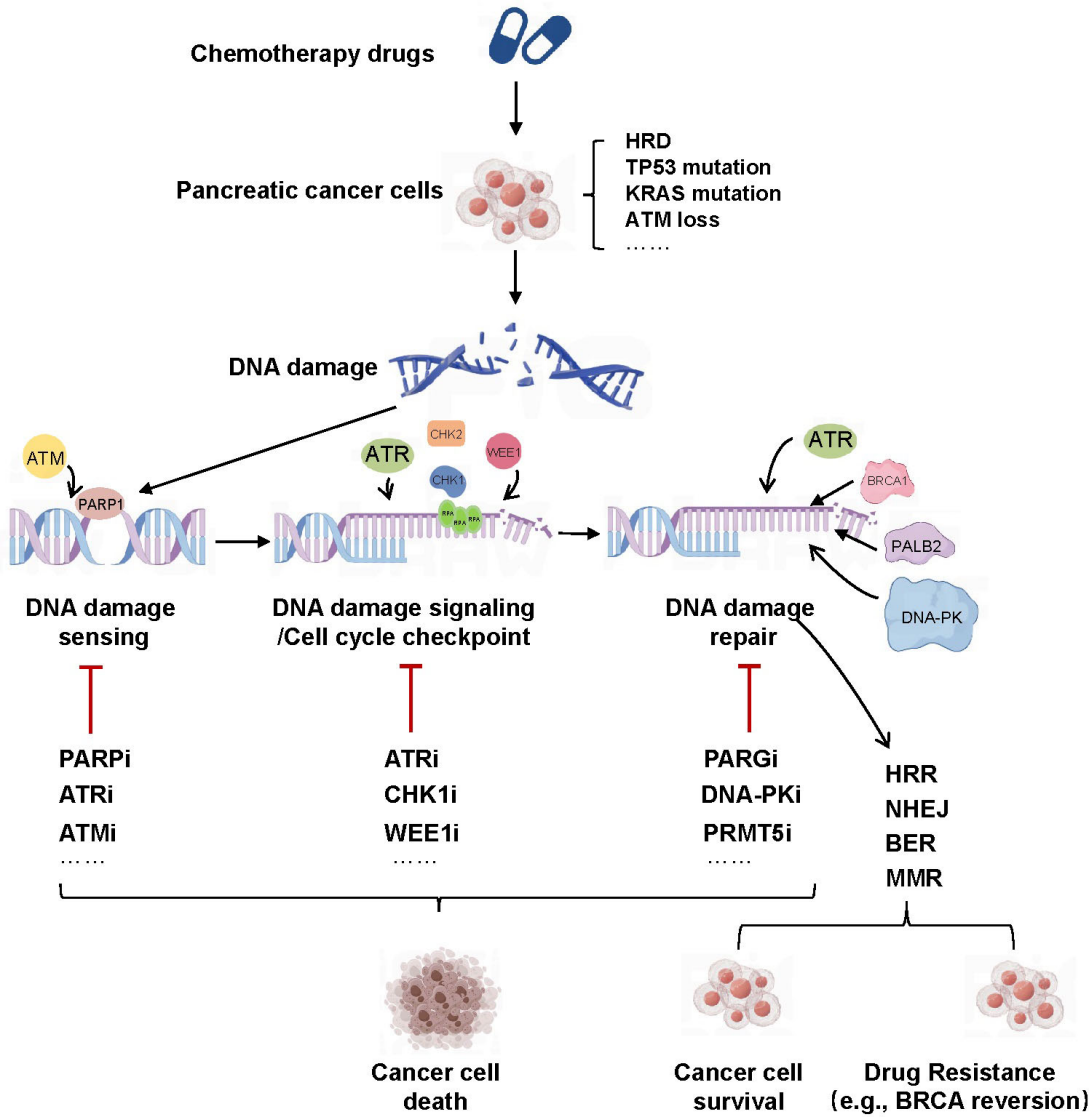
Schematic representation of the DDR pathway and key therapeutic targets in pancreatic cancer.

In pancreatic cancer, DDR-related gene alterations are mostly found in the HRR pathway. These changes destabilize the tumor genome and provide a molecular basis for targeted therapies (**Figure 1**). BRCA1 and BRCA2 are the most studied and most important HRR genes in pancreatic cancer. About 4–7% of patients carry germline BRCA1/2 mutations, and somatic mutations also account for a substantial proportion of cases (32, 33). The BRCA1 and BRCA2 proteins play central roles in HRR, responsible for damage site recognition, DNA end resection, and RAD51 recruitment and loading. If these proteins do not work, HRR is badly damaged. So the cells become very sensitive to PARP inhibitors and platinum chemotherapy. PALB2 is a bridge protein that links BRCA1 and BRCA2. Approximately 1–3% of pancreatic cancer patients have germline PALB2 mutations, which similarly cause homologous recombination deficiency (HRD) (34–36). Clinical studies have confirmed that patients carrying such mutations can benefit from PARP inhibitor therapy, with clinical significance analogous to that of BRCA mutations (37). The ATM gene serves as the primary sensor for DNA double-strand breaks. Germline or somatic mutations/deletions in ATM are found in approximately 5–10% of pancreatic cancer cases (38, 39). Although the loss of ATM function does not directly equate to canonical HRD, it severely impairs the activation of downstream HR repair signaling—such as through the phosphorylation of BRCA1 and CHEK2—resulting in a state of HRD or impaired HR capacity (40). This state makes tumor cells more susceptible to ATR inhibitors or PARP inhibitors, especially in specific combination strategies. Studies indicate that individuals with germline pathogenic ATM variants have an increased lifetime risk of pancreatic cancer, and work by ZH et al. suggests that deleterious ATM mutations may correlate with a better prognosis for pancreatic cancer patients (41, 42). ATR is a sensor that detects problems during DNA replication. ATR mutations themselves are uncommon, but ATR function can be altered by signals from other proteins, such as when ATM is defective. Therefore, blocking the ATR−CHK1 pathway offers an important therapeutic strategy for tumors with ATM defects and for tumors experiencing high replication stress (43). One study found that methylation of the FAM110C gene reduces genome stability, making pancreatic cancer cells dependent on the ATR−CHK1 pathway to manage replication stress and survive (44).

Other mutations—in genes such as CHEK2, the RAD51 family, and components of the Fanconi anemia pathway—add to the broader spectrum of HR−related gene alterations in pancreatic cancer (35, 45). In addition to well-studied DDR-related genes, new targets such as POLQ have attracted attention. POLQ (DNA polymerase theta) participates in DNA double-strand break repair through the error-prone microhomology-mediated end joining (MMEJ) pathway (46, 47). Pancreatic cancer cells with HRD rely on the POLQ-mediated MMEJ pathway to repair DNA damage and maintain survival. Therefore, blocking POLQ function disrupts this compensatory repair mechanism and leads to cell death, reflecting the concept of synthetic lethality. The study by Samnotra et al. is evaluating the clinical potential of combining a POLQ inhibitor with a PARP inhibitor, but the results have not yet been reported (48). **Table 1** summarizes the main mutated genes that cause DDR defects in pancreatic cancer and lists their details.

**Table 1:** Key DDR gene alterations in pancreatic cancer.

Citation	Gene category	Gene name	Key mutation type /frequency	Functional role in DDR pathway	Primary clinical & therapeutic implications
(49)	Core HRR Genes	BRCA1	Germline mutations ~1-2%; somatic mutations ~0.5-1%.	Damage recognition, DNA end resection, RAD51 recruitment.	Defines the classical HRD phenotype; confers high sensitivity to PARP inhibitors and platinum-based chemotherapy.
(50, 51)		BRCA2	Germline mutations ~3-5% (most common germline HRR mutation in PDAC).	RAD51 loading and functional regulation.	Along with BRCA1, serves as the strongest biomarker for predicting response to PARP inhibitors.
		PALB2	Germline mutations ~1-3%.	Acts as a bridge connecting BRCA1 and BRCA2.	Functionally equivalent to a “BRCA-like” mutation, leading to HRD and sensitivity to PARP inhibitors.
(52, 53)	HRR-Related Signaling Genes	ATM	Germline/somatic mutations or loss ~5-10%.	Primary sensor for DNA double-strand breaks; phosphorylates and activates downstream repair proteins.	Results in HRD; confers sensitivity to ATR inhibitors and may enhance sensitivity to PARP inhibitors.
(53, 54)		ATR, CHEK2	Mutations are relatively rare (<2%).	Transduce replication stress and DNA damage signals to activate cell cycle checkpoints.	Theoretically sensitive to ATR/CHK1 inhibitors; potential targets for combination therapy.
(55, 56)	Fanconi Anemia Pathway Genes	FANCA, FANCC, FANCG, etc.	Combined germline mutation frequency ~3-4%.	Involved in repairing DNA interstrand crosslinks, functioning in concert with the HR pathway.	May contribute to genomic instability and potentially influence response to DNA crosslinking agents (e.g., platinum drugs).
(57, 58)	HR Effector Genes	RAD51C, RAD51D, etc.	Mutation frequency is low (~1-2%).	Core catalytic components of HR repair, mediating DNA strand invasion and repair synthesis.	Directly impair HR function; may predict sensitivity to PARP inhibitors.
(46, 47)	Emerging/Alternative Repair Gene	POLQ	Mutations are uncommon, but frequently compensatorily overexpressed in HRD tumors.	Mediates error-prone MMEJ.	Serves as a “backup” repair pathway in HR-deficient tumors; a highly promising novel synthetic lethal target.

Synthetic lethality provides a key concept for targeted therapy of pancreatic cancer (59, 60). Its core idea is that breaking two genes or pathways at the same time kills the cell, while breaking only one leaves it alive (61). In cancer treatment, we apply this concept precisely: tumor cells already harbor one break—a gene defect—and we introduce a drug that causes a second break in a related pathway, killing the tumor cells while largely sparing normal cells. In pancreatic cancer, this works well because some tumors already have HRD. A subset of patients carry germline or somatic mutations in genes such as BRCA1, BRCA2, and PALB2, which disable HR (62). That disabling constitutes the first break in these tumor cells, and this HRD state makes the tumor cells vulnerable when repairing DNA double−strand breaks.

DDR inhibitors target a specific vulnerability in pancreatic cancer cells: the HRD state. PARP inhibitors block the backup repair path that these cells need to survive. By doing this, they turn HRD into a way to kill the cells (61). Clinical studies validated this concept, leading to the approval of drugs such as olaparib for pancreatic cancer patients with germline BRCA mutations. This approval marked a significant step, bringing pancreatic cancer into the era of molecularly stratified targeted therapy.

## 3 DDR inhibitors in pancreatic cancer: classification, mechanisms, and clinical progress

DDR inhibitors offer a promising approach for treating pancreatic cancer by targeting the tumor’s genomic instability. As we learn more about molecular subtypes such as HRD, the application of DDR inhibitors has expanded beyond PARP inhibitors to include drugs targeting other key nodes like ATR, CHK1, and WEE1 (63). PARP inhibitors such as olaparib work through synthetic lethality and have become a standard maintenance treatment for patients with germline BRCA mutations, shifting therapy from chemotherapy to precision targeted therapy. ATR inhibitors target conditions such as HRD caused by ATM loss, while CHK1 and WEE1 inhibitors block cell cycle checkpoints and synergize with chemotherapy; early studies suggest they may help overcome resistance and improve treatment efficacy. Collectively, these advances demonstrate that DDR inhibitors are transforming pancreatic cancer treatment by matching the tumor’s molecular characteristics. In this section, we will examine different DDR inhibitors according to their targets and mechanisms, key clinical data, and the latest research.

### 3.1 PARP inhibitor

PARP inhibitors remain the only class of DDR inhibitors approved for pancreatic cancer, and their success validates the concept of synthetic lethality. They kill tumor cells by interfering with DNA repair through a well−defined mechanism. Under normal conditions, PARP1 detects and repairs single−strand DNA breaks. PARP inhibitors such as olaparib both block PARP1’s repair activity and trap PARP1 on the DNA, creating a physical obstacle. When the cell replicates its DNA, this obstacle causes replication fork collapse, leading to double−strand breaks. Cells with functional HRR can repair these breaks, but tumor cells with defective HRR—due to mutations in genes such as BRCA1/2 or PALB2—accumulate damage, triggering synthetic lethality and cell death.

Built on a clear concept, PARP inhibitors have become one of the most widely used and important targeted drugs for precision treatment of pancreatic cancer (64, 65). Olaparib is a good example. Final analysis of the POLO trial showed that, although overall survival (OS) did not reach statistical significance, the treatment extended the chemotherapy-free interval and improved survival rates (66). Based on these clinically meaningful benefits, olaparib remains approved as maintenance therapy for patients with advanced pancreatic cancer after first-line platinum-based chemotherapy (67). It also marks the start of targeted therapy based on molecular traits in pancreatic cancer. Other PARP inhibitors, including niraparib, talazoparib, and rucaparib, have also shown promise in preclinical and early clinical studies (68–70). While all PARP inhibitors share the core mechanism of inducing synthetic lethality in an HRD context by inhibiting PARP catalytic activity, trapping cytotoxic PARP-DNA complexes, and disrupting base excision repair, their key distinctions lie in their differential capacities for PARP protein trapping and enzyme inhibition (71, 72). Studies have established a relative ranking of PARP-trapping potency among these agents: talazoparib > olaparib ≈ niraparib > rucaparib > veliparib (73, 74). The following table systematically outlines the clinical trial advancements of key PARP inhibitors (**Table 2**).

**Table 2:** PARP inhibitors in clinical development for pancreatic cancer.

Citation	Drug name	Development status	Key clinical trial progress
(66)	Olaparib	Approved	Approved indication: Based on the POLO Phase III trial, it is indicated for the maintenance therapy of patients with metastatic pancreatic cancer harboring a germline BRCA mutation who have not progressed following first-line platinum-based chemotherapy.
(75)	Niraparib	Phase II	It is evaluating its use as monotherapy or in combination with immunotherapy for advanced pancreatic cancer harboring germline BRCA or PALB2 mutations.
(76)	Talazoparib	Preclinical/Early Clinical	It has demonstrated potent activity in preclinical models of HRD pancreatic cancer, and combination strategies based on it are a major research focus.
(77)	Rucaparib	Phase II (Basket Trial)	A Phase II basket trial evaluated its antitumor activity in advanced solid tumors, including pancreatic cancer, with germline or somatic BRCA1/2 or PALB2 mutations, showing preliminary activity.
(37, 78)	Veliparib	Phase II	It combined with gemcitabine/cisplatin did not improve outcomes much over chemotherapy alone in a Phase III study, and caused more blood side effects. In other Phase II study, veliparib with FOLFOX showed promising activity, particularly in platinum−naïve patients with a pathogenic HR−DDR mutation.
(79)	Fluzoparib	Phase Ib/II	First-line combination therapy: An Ib/II Phase study (NCT04228601) is evaluating its combination with mFOLFIRINOX chemotherapy as first-line treatment for locally advanced or metastatic pancreatic cancer, followed by Fluzoparib monotherapy maintenance.

Despite the success of the POLO trial, real−world implementation of its findings faces several challenges (80). First, the POLO trial applied strict eligibility criteria. It required patients to complete at least 16 weeks of platinum−based chemotherapy without disease progression, maintain good performance status, and have adequate organ function. These criteria exclude many patients in routine practice. In real−world settings, patients with germline BRCA−mutant pancreatic cancer often have poorer performance status or more comorbidities. Some also progress early on first−line chemotherapy. Thus, they do not qualify for olaparib maintenance (67, 80). Second, limited access to genetic testing creates another barrier. Guidelines recommend universal germline testing for all pancreatic cancer patients, but many regions still have low testing rates. Consequently, clinicians miss opportunities to identify eligible patients (81, 82). Third, real−world toxicity profiles may differ from trial data. Fatigue, anemia, and nausea commonly occur. These side effects can force dose reductions or treatment discontinuation, particularly in elderly or frail patients (80, 83). Fourth, we still lack clear evidence of olaparib’s benefit outside the maintenance setting. For example, patients who never received prior platinum chemotherapy, or those with somatic (not germline) BRCA mutations, have not shown clear benefit. Current evidence does not support routine use in these subgroups (84). Together, these factors reveal a substantial gap between the efficacy demonstrated in the POLO trial and the effectiveness we achieve in everyday clinical practice.

PARP inhibitors still have clinical limitations because only a small group of patients can benefit. Currently, good responses occur mostly in patients with germline BRCA1/2 mutations, who represent only about 4–7% of all pancreatic cancer cases. For patients with other HRR gene mutations (such as ATM or PALB2) or without clear HRD markers, PARP inhibitors alone do not work well. Therefore, expanding the eligible patient population remains a key challenge.

### 3.2 ATR inhibitors

The accumulation of replication stress makes HRD tumors dependent on ATR. ATR inhibitors combined with chemotherapy or other targeted agents have shown efficacy in several cancer types (85). In pancreatic cancer, these inhibitors also represent a new option worth considering. ATR inhibitors target replication stress and specific gene defects, and their use in pancreatic cancer operates through two main mechanisms. First, they target HRD—such as in ATM loss—to induce synthetic lethality. In cancer cells lacking ATM, the DNA damage signal does not transmit properly, making these cells highly dependent on ATR to maintain replication fork stability and manage replication stress (86, 87). ATR inhibitors block this key pathway, causing synthetic lethality in ATM−deficient cells and killing tumor cells in a targeted manner. Second, they synergize with chemotherapy to enhance its effectiveness. Pancreatic cancer cells often experience high replication stress; ATR inhibitors disable cell cycle checkpoints, so tumor cells with DNA damage cannot repair it. This exacerbates chemotherapy−induced damage and helps overcome drug resistance (88).

ATR inhibitors do not kill cells randomly; their efficacy depends heavily on the tumor cell’s intrinsic state (such as ATM loss) or extrinsic pressure (e.g., chemotherapy exposure). Their value lies in transforming these two scenarios into therapeutic advantages, representing an emerging, mechanism−driven precision strategy in pancreatic cancer treatment. Currently, several ATR inhibitors are under investigation in pancreatic cancer at various stages—from preclinical to clinical research. They primarily exert their effects by targeting replication stress and specific DNA repair defects in tumor cells, as summarized in **Table 3**.

**Table 3:** ATR inhibitors in clinical development for pancreatic cancer.

Citation	Drug name	Development status	Key research evidence and characteristics
(89)	Alnodesertib (ART0380)	Phase II	Currently in clinical trials for pancreatic cancer patients with ATM loss or low expression in the second-line setting. When combined with low-dose irinotecan, it achieved an objective response rate of 50% in ATM-negative patients with advanced solid tumors (including pancreatic cancer).
(90, 91)	Elimusertib (BAY 1895344)	Phase I	A Phase I trial initiated in 2021 is evaluating its combination with irinotecan or topotecan for the treatment of advanced solid tumors, including metastatic pancreatic cancer. Preclinical data suggest potential efficacy in tumors with high replication stress, such as pancreatic cancer.
(92)	Ceralasertib (AZD6738)	Preclinical/Early Clinical	Multiple preclinical studies indicate that when combined with olaparib, it can effectively inhibit the growth of pancreatic cancer with proficient homologous recombination repair, showing potential to overcome PARP inhibitor resistance.
(93)	VE-822 (VX-970)	Preclinical/Phase II	One of the earlier ATR inhibitors to enter clinical development. Preclinical studies have confirmed its ability to sensitize pancreatic cancer cells to various chemotherapeutic agents, such as gemcitabine.
(94)	ATR PROTAC (proteolysis-targeting chimeras)	Preclinical Discovery Stage	This emerging class of PROTAC is designed to directly degrade the ATR protein rather than merely inhibit its activity. Effective degradation of ATR protein has been achieved in pancreatic cancer cell lines, representing a frontier exploration direction.

In pancreatic cancer treatment, ATR inhibitors share significant commonalities because they target the same molecule and core mechanism, yet they also exhibit key differences in chemical structure, clinical development strategies, and applicable patient populations. Their common target (ATR) and core mechanism (combination−mediated sensitization) define them as a class of “team−player” drugs that amplify the efficacy of existing therapies. Their differences primarily manifest in their modes of action and choice of combination partners. However, despite theoretical promise, ATR inhibitors face major challenges in clinical application. In recent years, PROTAC technology has provided a new tool for targeting ATR protein (94). This may more durably block pathway activity and overcome resistance mutations. Preliminary anti-proliferative effects have been observed in pancreatic cancer cell lines. Future clinical investigation is anticipated. Moving downstream along the same pathway, CHK1 and WEE1 constitute two additional nodes in the replication stress response.

### 3.3 CHK1 and WEE1 inhibitors

CHK1 inhibitors and WEE1 inhibitors are two drug classes that both target key cell cycle checkpoints within the DDR pathway. They block DNA repair in tumor cells after damage, causing cells to enter the next cell cycle Phase with unrepaired damage. This enhances the efficacy of chemotherapy, particularly gemcitabine, aiming to overcome drug resistance and improve treatment outcomes (95–97). Their clinical applications are similar, but their developmental stages differ. **Table 4** summarizes these differences.

**Table 4:** CHK1 and WEE1 inhibitors in clinical development for pancreatic cancer.

Citation	Inhibitor class	Drug name (code)	Development sstatus	Key clinical research progress and findings
(98)	CHK1 Inhibitors	LY2603618	Phase II	A Phase II study combining it with gemcitabine showed no significant improvement in patient overall survival compared to gemcitabine alone, and the study did not meet its primary endpoint
(99)		LY2880070	Phase I	When combined with low-dose gemcitabine for the treatment of advanced pancreatic cancer, no objective response was observed in patients, indicating limited clinical efficacy. Interestingly, organoid models established from patient biopsies were highly sensitive to the combination regimen, suggesting a translational gap between preclinical models and clinical efficacy in humans
(100)		Prexasertib	Preclinical/Early Clinical	Its combination with BRD4770 demonstrated a synergistic inhibitory effect on replication-related phenomena, including cell growth, DNA synthesis, S-Phase cell cycle progression, and DNA damage signaling, ultimately leading to efficient induction of cell death
(101)	WEE1 Inhibitors	Azenosertib (ZN-c3)	Phase II (Ongoing)	A Phase II trial (NCT06015659) is now testing this drug with gemcitabine. It is for advanced pancreatic cancer patients whose cancer got worse after first−line FOLFIRINOX. This is one of the most advanced clinical studies in the field.
(102)		Adavosertib (AZD1775)	Preclinical Research	Multiple preclinical studies have shown that it can effectively sensitize pancreatic cancer cells to chemotherapeutic agents such as gemcitabine.

CHK1 inhibitors act downstream of ATR and aim to block the S and G2 checkpoints. However, a Phase II study combining the CHK1 inhibitor LY2603618 with gemcitabine failed to extend overall survival in patients with advanced pancreatic cancer and did not meet its primary endpoint (98). This result suggests that blocking CHK1 alone may be insufficient to achieve the desired effect in the complex microenvironment of human tumors. WEE1 inhibitors work differently: they target the G2/M checkpoint activated after DNA damage. This target matters in pancreatic cancer because over half of cases carry TP53 mutations, which disable the G1 checkpoint; consequently, cancer cells depend on the G2/M checkpoint—controlled by WEE1—for survival (103–105). This mechanism provides the theoretical foundation for WEE1 inhibitor efficacy. For instance, a 2019 study demonstrated that the WEE1 inhibitor Adavosertib (AZD1775) sensitized pancreatic cancer tumors to gemcitabine (102).

Both CHK1 and WEE1 inhibitors remain in early to mid−Phase exploratory clinical stages and have not yet been approved, but current data show that WEE1 inhibitors appear more promising for chemosensitization.

### 3.4 ATM/DNA-PK inhibitors

ATM and DNA−PK inhibitors critically target the DNA double−strand break repair pathway, though their clinical development in pancreatic cancer remains at an early stage (preclinical to Phase I/II). They function not as monotherapy but as radiosensitizers or in combination with other targeted agents such as PARP inhibitors, aiming to overcome resistance and enhance tumor cell killing in HRD tumors (53, 106). The most promising direction currently is the “PAD” combination strategy, which simultaneously inhibits PARP, ATR, and DNA−PK. This strategy delivers a multi−node, synergistic attack on the DNA repair network of HRD tumors (e.g., those with ATM or BRCA mutations). Mechanistically, PARP inhibitors induce DNA damage and trap PARP proteins on DNA; ATR inhibitors disrupt checkpoints that respond to replication stress; and DNA−PK inhibitors (e.g., CC−115) block NHEJ, the ultimate backup pathway for double−strand break repair. The synergistic action of all three agents completely abolishes tumor cell repair capacity, leading to irreversible DNA damage accumulation and cell death. A pivotal 2025 preclinical study further optimized this regimen by replacing olaparib with talazoparib (a potent PARP inhibitor), establishing the “PAD_tal_” (talazoparib−based PAD) regimen. This optimized regimen was effective against a broad spectrum of HRD genotypes in pancreatic cancer—including deficiencies in ATM, BRCA1, BRCA2, and PALB2—while demonstrating favorable tolerability *in vivo*. These findings provide a strong rationale for future clinical translation (76).

Currently, ATM/DNA-PK inhibitors have not yet entered routine clinical use for pancreatic cancer. However, they still show considerable potential as key components of combination strategies—especially when combined with radiotherapy or PARP inhibitors (see **Table 5**). It should also be noted that no ATR, WEE1, or DNA-PK inhibitor has yet been approved for standard treatment of pancreatic cancer to date. This fact highlights the gap between theoretical promise and actual clinical evidence.

**Table 5:** ATM/DNA-PK inhibitors in clinical development for pancreatic cancer.

Citation	Inhibitor class	Drug name (code)	Development status	Key clinical research progress and findings
(107, 108)	ATM Inhibitor	AZD0156	Preclinical/Early Clinical Research	Preclinical studies indicate that simultaneous inhibition of PARP and ATM enhances the sensitivity of pancreatic cancer to irreversible electroporation (IRE) therapy;AZD0156 enhances radiotherapy-induced antitumor immune responses and sensitizes pancreatic cancer to immunotherapy.
(109)	DNA-PK Inhibitor	AZD7648	Preclinical Research	In xenograft and PDX models, AZD7648 enhanced the efficacy of olaparib across a range of doses and schedules, achieving sustained tumor regression and providing a clear rationale for its clinical investigation.
(106)	ATM/DNA-PK Dual Inhibitor	XRD-0394	Phase I Clinical Trial (Completed, NCT05002140)	A dual inhibitor of ATM and DNA-PK, designed to enhance the efficacy of radiotherapy or PARP inhibitors; a Phase I study in combination with palliative radiotherapy has been completed.

### 3.5 Emerging targets

In addition to the extensively studied inhibitors targeting PARP, ATR, CHK1/WEE1, and ATM/DNA−PK, recent years have seen several novel targets emerge within the DNA damage repair pathway of pancreatic cancer. These emerging targets function primarily through epigenetic regulation, novel synthetic lethal mechanisms, or expansion of the HRD−related protein network, and several candidates have entered preclinical or early−stage clinical development. The emergence of these novel inhibitors signals three major evolving trends in DDR−targeted therapy:

1. PRMT5 is a protein arginine methyltransferase that catalyzes symmetric dimethylation of histones and various regulatory proteins, affecting critical oncogenic processes such as epithelial−mesenchymal transition, MYC signaling, and glycolysis (110, 111). Some types of pancreatic cancer are sensitive to PRMT5 inhibition (112). Mechanistically, PRMT5 inhibition depletes RPA, leading to DNA damage accumulation and creating a synthetic lethal interaction with gemcitabine (112). PRMT5 blockers also work with PARP inhibitors; this combination extended survival in mice and worked in both BRCA−mutant and wild−type patient−derived xenograft (PDX) models (113). This strategy of epigenetically inducing DDR deficiencies offers a novel approach to expand the eligible patient population for PARP inhibitors.
2. Poly(ADP-ribose) glycohydrolase (PARG) inhibitors block the removal of PAR chains, leading to excessive PARP1 activity and replication fork instability. Preclinical studies show that PARG inhibitors sensitize cells to radiation damage and slow replication forks. In cancer cells resistant to PARP inhibitors, these drugs work about as well as nedaplatin (114). Targeting other HR pathway components—such as BARD1 and PARG—may open new therapeutic avenues for treating tumors (115–117).
3. HuR is an RNA−binding protein that translocates from the nucleus to the cytoplasm upon DNA damage. There, it helps stabilize specific mRNAs, such as WEE1, thereby playing a role in DNA repair (118). Silencing HuR makes pancreatic cancer cells more sensitive to DNA−damaging drugs like oxaliplatin and PARP inhibitors, positioning HuR as a potential target to overcome chemotherapy and PARP inhibitor resistance (118).

It is noteworthy that most of these novel inhibitors are currently in preclinical or early-stage clinical development and have not yet reached regulatory approval, as summarized in the table below (**Table 6**). Nonetheless, they represent important frontiers in precision oncology for pancreatic cancer and other malignancies, paving the way to expand the reach of DDR−targeted therapies.

**Table 6:** Emerging targets in clinical development for pancreatic cancer.

Citation	Inhibitor class	Representative targets/drugs	Developmental stage	Key clinical research progress and findings
(119)	PRMT5 Inhibitors	AMG 193	Phase I	A Phase 1b study (NCT06360354) is evaluating the safety, tolerability, and efficacy of AMG 193 in combination with chemotherapy (gemcitabine + nab-paclitaxel or mFOLFIRINOX) in patients with advanced pancreatic cancer harboring MTAP deficiency.
–	PARG Inhibitors	DAT-2645/IDE161	Preclinical	Currently, there are no clinical trial data available for this small-molecule PARG inhibitor in pancreatic cancer.
(120)	Targeting BARD1	No specific drug candidates named yet	Target discovery and mechanistic studies	Currently, BARD1 has been incorporated into germline mutation testing panels for pancreatic cancer.
(121)	Targeting HuR	Multiple preclinical small-molecule inhibitors	Target discovery and mechanistic studies	HuR knockdown significantly reduces the proliferation, migration, and invasion capabilities of PDAC cells and suppresses tumor growth in mouse xenograft models

## 4 Combination therapeutic strategies of DDR inhibitors in pancreatic cancer

The combination strategies for DDR inhibitors in pancreatic cancer used to be just chemotherapy plus a targeted drug, but now they are more than that. Based on the latest preclinical and clinical evidence, the following are the current major combination strategies and their detailed mechanisms are summarized below.

### 4.1 Combination with chemotherapy

This core principle underpins the clinical application of DDR inhibitors and has been most extensively validated with PARP inhibitors. Platinum agents or gemcitabine directly induce DNA damage or trigger replication stress in tumor cells, killing them. DDR inhibitors exacerbate this damage by blocking DNA repair pathways, such as PARP−mediated BER and ATR−mediated checkpoint regulation (25, 95, 122, 123). A recent study demonstrated that the ATR inhibitor BAY 1895344 combined with oxaliplatin slows the growth of platinum−resistant pancreatic cancer cells in both laboratory and animal models (124). Therefore, this combination may offer an effective strategy to overcome chemotherapy resistance.

### 4.2 PAD regimen

The PAD regimen is a triple combination targeting the DDR pathway, consisting of a PARP inhibitor, an ATR inhibitor, and a DNA-PK inhibitor. It is a promising mechanism-driven strategy for HRD pancreatic cancer. The core idea is to attack the tumor’s DNA repair system at multiple points (53). In this regimen, the PARP inhibitor damages DNA and traps PARP on the DNA; the ATR inhibitor abrogates the checkpoints that respond to replication stress; and the DNA-PK inhibitor blocks the last repair path (NHEJ) for double−strand breaks (71, 95, 125). Together, these actions shut down the tumor’s repair network, leading to accumulation of damage and cell death.

The concept of PAD builds on earlier work (53). The original PAD regimen uses olaparib as the PARP inhibitor. In a 2025 preclinical study, an optimized version named PAD_tal_ was introduced, replacing olaparib with the more potent talazoparib. This new regimen was effective in ATM-deficient models and other HRD types, including BRCA1, BRCA2, and PALB2 defects (76). PAD_tal_ also showed good *in vivo* tolerability, supporting its clinical translation (76).

### 4.3 Emerging dual-target inhibitors

This class represents a new “combination” strategy by integrating two therapeutic targets into one molecule. Nesuparib, a dual-target inhibitor, inhibits both PARP and tankyrase. Tankyrase inhibition disrupts the WNT and Hippo signaling pathways, both critical for HRR (126, 127). Consequently, Nesuparib induces a “BRCAness” state, allowing tumors without BRCA mutations to remain sensitive to PARP inhibitors and thereby substantially expanding the potential beneficiary population (128). A Phase Ib study is now testing nesuparib with mFOLFIRINOX or with gemcitabine plus nab−paclitaxel (128).

### 4.4 Sequential regimen

To overcome drug resistance from PARP inhibitors or platinum chemotherapy, researchers have proposed the concept of sequential therapy. A study published in the *British Journal of Cancer* demonstrated that administration of the ATR inhibitor ceralasertib before the PARP inhibitor olaparib effectively overcomes resistance models. Conversely, in DDR-deficient models, olaparib treatment followed by ceralasertib yielded superior outcomes (92). Therefore, timing drugs according to tumor traits may improve treatment efficacy and reduce the side effects often associated with concurrent administration.

### 4.5 Combination with immunotherapy

Pancreatic cancer is a “cold tumor” that does not respond well to immune checkpoint inhibitors (129). DDR inhibitors, particularly those targeting ATM/ATR, can activate the cGAS-STING pathway by inducing DNA damage. That leads to type I interferon and brings in CD8+ T cells, so the tumor changes from “cold” to “hot” (108). For example, the ATM inhibitor AZD0156 enhances radiation−induced immune activation and sensitizes pancreatic cancer to immunotherapy such as anti−PD−L1 (130). Similarly, in BRCA1-deficient breast cancer models, the PARP inhibitor olaparib induces CD8+ T cell infiltration and activation. Adding a STING agonist further boosts STING pathway activation, leading to changes in the tumor microenvironment (131). These findings provide theoretical rationale for combining DDR inhibitors with immunotherapeutic strategies.

## 5 Current challenges

DDR inhibitors, especially PARP inhibitors, have opened up new targeted treatment options for pancreatic cancer patients with HRD. But using them in the clinic still comes with many problems. These problems also point to what we need to focus on next.

### 5.1 Drug resistance

**Figure 2:**
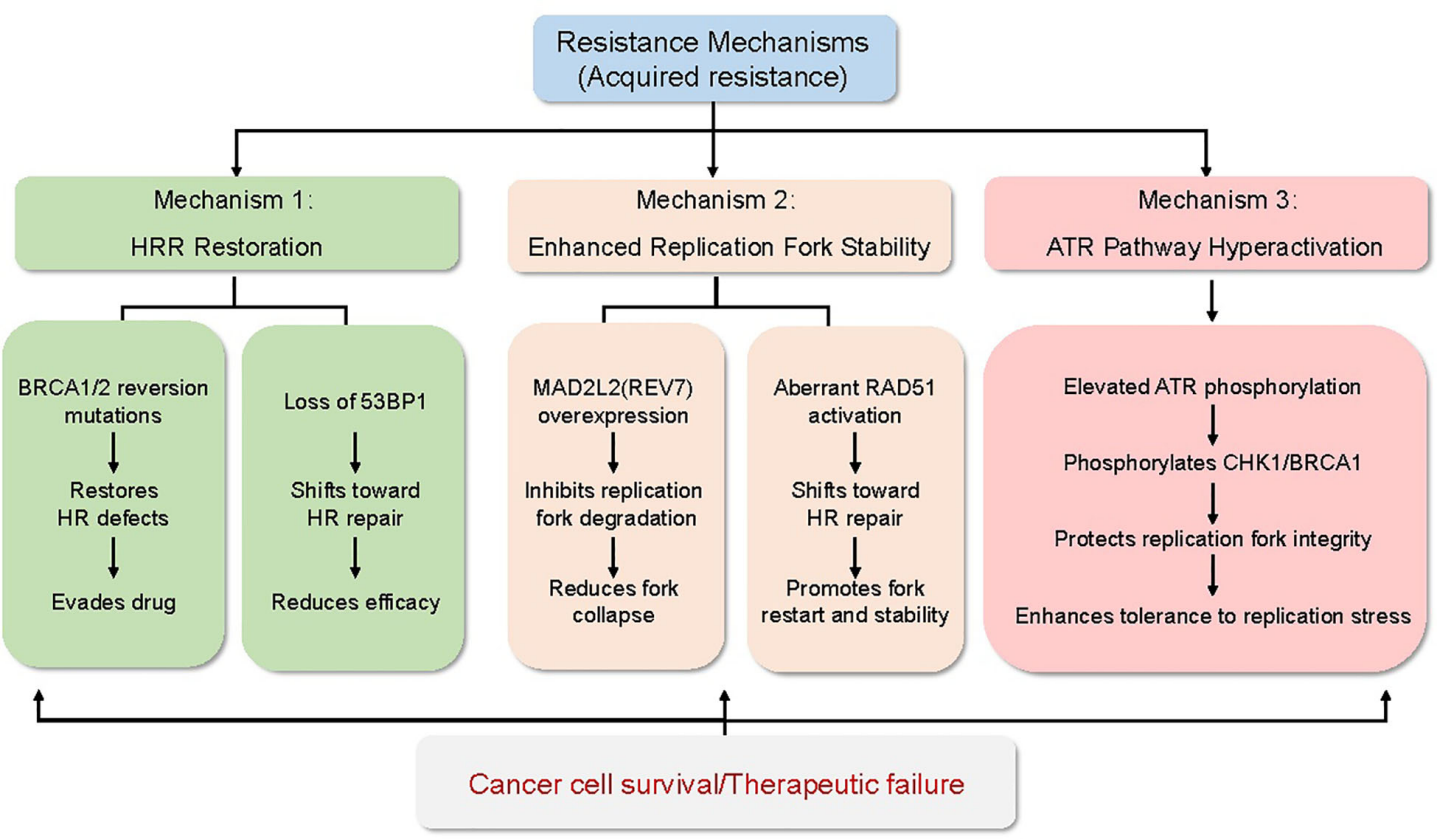
Mechanisms of drug resistance to DDR inhibitors in pancreatic cancer.

One of the main challenges for DDR inhibitors in clinical use is the development of drug resistance, which is mainly divided into primary and acquired resistance. Both types reduce long-term treatment efficacy and may even lead to complete failure. Many studies show that acquired resistance to DDR inhibitors is closely linked to transcriptomic changes in multiple pathways, including cell cycle checkpoints, metabolism, DNA damage response, apoptosis, and replication stress. In pancreatic cancer, although the PARP inhibitor olaparib has been approved for patients with germline BRCA mutations, acquired resistance rapidly emerges and severely limits its long-term efficacy (132). Three common mechanisms of resistance have been clearly identified (**Figure 2**):

1. HRR is the core target of DDR inhibitors for killing tumors (133). Resistance can occur through restoration of HR function. A typical mechanism is BRCA reversion mutations: secondary mutations in BRCA1/2 fix the HR defect caused by the original mutation, allowing tumor cells to evade the drug (134). In pancreatic cancer, case reports have shown BRCA2 reversion mutations after olaparib treatment, leading to clinical resistance (135). In addition, loss of 53BP1 can shift cells toward HR repair, reducing the efficacy of DDR inhibitors (136).
2. DDR inhibitors induce accumulation of DNA damage in tumor cells by disrupting replication fork stability. Tumor cells can develop resistance by enhancing replication fork stability, for example through overexpression of MAD2L2 (REV7), which inhibits replication fork degradation (137, 138). Aberrant activation of RAD51 promotes replication fork restart and stability, thereby reducing drug killing efficiency (139). In pancreatic cancer cell lines, upregulating MAD2L2 expression significantly reduces platinum-induced replication fork collapse, leading to acquired resistance (140).
3. ATR is a core kinase in the replication stress response. It phosphorylates downstream substrates (such as CHK1 and BRCA1) to protect replication fork integrity. In resistant cells, phosphorylation levels of ATR and downstream signaling are often elevated, enhancing tumor tolerance to replication stress (141). In preclinical models of pancreatic cancer, combining ATR inhibition partially reverses PARP inhibitor resistance, further confirming the key role of this pathway in pancreatic cancer resistance (92).

### 5.2 Limited beneficiary population

PARP inhibitors work well mostly in patients with germline BRCA1/2 mutations, who represent only about 4–7% of all pancreatic cancer cases. Although roughly 20% of pancreatic cancer patients carry mutations in HRR−related genes, only those with germline BRCA1/2 mutations—about 5% of all cases—qualify for approved olaparib treatment. For patients with other HRR gene mutations (such as ATM or PALB2) or without clear HRD markers, DDR inhibitors alone do not work well.

### 5.3 Dose-limiting toxicities

Hematologic toxicity—including anemia, neutropenia, and thrombocytopenia—represents the most prevalent dose−limiting toxicity of DDR inhibitors. Early clinical trials of ATR inhibitors demonstrate that intermittent dosing regimens improve tolerability. Although combining multiple DDR inhibitors concurrently may improve therapeutic efficacy, it markedly elevates the risk of cumulative toxicity.

### 5.4 Barrier effect of dense stroma

In pancreatic cancer, dense fibrotic stroma accounts for up to 80% of tumor volume. It elevates interstitial hydrostatic pressure and compresses blood vessels, severely blocking delivery of small-molecule inhibitors targeting the DDR and preventing them from reaching effective concentrations (142–146). Meanwhile, cancer-associated fibroblasts (CAFs) in the stroma directly lower the sensitivity of tumor cells to DDR inhibitors. As a result, cell cycle checkpoint blockade fails, and the replication stress response is impaired. This reduces the killing effect of small-molecule DDR inhibitors (24).

### 5.5 Uneven clinical development

With the exception of PARP inhibitors, inhibitors targeting other DDR nodes (e.g., ATM, ATR, CHK1, WEE1) remain predominantly in preclinical or early-Phase clinical development and are far from regulatory approval. The clinical development of some ATR inhibitors has been modified owing to efficacy or strategic considerations.

## 6 Future perspective

Given the challenges of DDR inhibitors in pancreatic cancer discussed above, future breakthroughs in this field will rely on coordinated progress in multiple directions.

### 6.1 New biomarkers

Developing biomarkers is key to expanding the benefiting population. In addition to existing germline BRCA mutation testing, new testing methods or biomarkers should be further promoted. For example, HRD score based on genomic scarring and functional assays detecting RAD51 foci formation. HRD score identifies patients with inactive HR function by recognizing “scars” left on the genome from DNA double-strand break repair (147). RAD51 testing, independent of mutation status, can identify patients likely sensitive to platinum agents and PARP inhibitors (148). Furthermore, studies have shown that understanding the association between DDR and replication stress in pancreatic cancer is crucial for developing a new biomarker-driven therapeutic strategy targeting both pathways (149). These new methods can identify patients with HR dysfunction even without clear HRR mutations, thereby expanding the potential beneficiaries of DDR-targeted drugs (such as PARP and ATR inhibitors) to a broader population.

### 6.2 Liquid biopsy

In the field of precision oncology, liquid biopsy holds promise as a new method for real-time monitoring of drug resistance. Liquid biopsy analyzes tumor-derived materials in body fluids (mainly ctDNA in blood) to detect BRCA reversion mutations and other HRR gene changes early, warning of drug resistance before radiological progression (150). Unlike traditional tissue biopsy, which requires surgery or needle puncture to obtain tumor tissue, liquid biopsy only needs peripheral blood or other body fluids. It is less invasive and highly repeatable, making it particularly suitable for dynamic monitoring during treatment.

### 6.3 Combination therapy with KRAS inhibitors

Combination therapy strategies need to expand to more diverse targets, particularly combinations with KRAS inhibitors. Given that over 90% of pancreatic cancers harbor KRAS mutations, and there is potential crosstalk between KRAS signaling and DDR (151). Exploring combinations of PARP or ATR inhibitors with KRAS inhibitors may extend the benefits of DDR-targeted therapy to patients without HRR mutations.

### 6.4 More rational trial design

Only a small fraction of pancreatic cancer patients carry specific HRR mutations (e.g., ATM, PALB2), making traditional single randomized trials nearly impossible. Therefore, clinical trial design needs to shift toward more efficient models. Basket trials can evaluate the efficacy of a given DDR inhibitor in pancreatic cancer and other solid tumors carrying different HRR mutations (including BRCA, PALB2, ATM, etc.). Platform trials can simultaneously enroll multiple combination regimens, dynamically adjust group allocation based on interim analysis results, thereby accelerating the selection of effective regimens while avoiding further investment in ineffective ones.

## 7 Conclusion

Pancreatic cancer is one of the deadliest cancers, and treatment options remain few. Because the tumor exhibits genomic instability and relies on DDR pathways for survival, DDR−targeted drugs represent a key step forward in precision treatment. PARP inhibitors have performed well in the clinic. Olaparib is now approved for pancreatic cancer with germline BRCA mutations, demonstrating that the concept of synthetic lethality works in this disease. Treatment has moved from non−selective chemotherapy to molecularly guided therapy, highlighting the importance of identifying DDR−related gene alterations (e.g., BRCA1/2, PALB2, ATM mutations) as predictive biomarkers. Beyond PARP inhibitors, new DDR−targeted agents—including ATR, WEE1, and DNA−PK inhibitors—help fill the gaps for patients who do not benefit from current treatments. For instance, ATR inhibitors have shown promise in preclinical and early clinical studies, especially in tumors with ATM loss or high replication stress, offering an option for patients with HRD who cannot use PARP inhibitors alone (89). WEE1 inhibitors act through a distinct mechanism by targeting a vulnerability in TP53−mutant pancreatic cancer, which accounts for more than half of all cases (152). These advances reflect a shift from one−size−fits−all treatment to molecularly stratified therapy, which matters greatly for a disease as heterogeneous as pancreatic cancer.

Combination strategies have expanded the clinical utility of DDR inhibitors. The PAD/PAD_tal_ triple regimen, dual−target inhibitors such as nesuparib, and sequential scheduling have helped address key limitations of single−agent use, including a narrow beneficiary population and acquired resistance. Furthermore, emerging targets such as PRMT5, PARG, and HuR—along with novel approaches like liquid biopsy and combinations with KRAS inhibitors—hold promise for expanding the reach of DDR−targeted therapies to more patients. There is also a growing link between DDR pathways and the immune system, providing a rationale for combining DDR inhibitors with immunotherapy.

DDR−targeted therapies still face several challenges before reaching their full clinical potential. Currently, only a small subset of patients can benefit, so we need to expand access. Some patients show primary resistance, while others acquire resistance after initial response. Overcoming drug resistance requires multidisciplinary approaches to study resistance mechanisms and develop rational drug combinations.

Looking ahead, DDR−targeted therapy for pancreatic cancer lies at the intersection of precision medicine, translational research, and innovative clinical trial design. Identifying new DDR−related targets like PRMT5, PARG, and HuR, as well as developing multi−target inhibitors, can open new therapeutic avenues and help overcome resistance. Ultimately, the success of DDR−targeted therapy in pancreatic cancer will depend on clarifying the crosstalk between genomic alterations, tumor heterogeneity, and the tumor microenvironment, and translating mechanistic insights into clinically actionable strategies to improve survival for patients with this devastating disease.

## Glossary

ATM: Ataxia telangiectasia mutated
ATR: Ataxia telangiectasia and Rad3-related
BARD1: BRCA1-associated RING domain protein 1
BER: Base excision repair
BRCA1: Breast cancer susceptibility gene 1
BRCA2: Breast cancer susceptibility gene 2
CAF: Cancer-associated fibroblast
cGAS: Cyclic GMP-AMP synthase
CHK1: Checkpoint kinase 1
CHK2: Checkpoint kinase 2
ctDNA: Circulating tumor DNA
DDR: DNA damage response
DNA-PK: DNA-dependent protein kinase
FOLFIRINOX: Folinic acid + fluorouracil + irinotecan + oxaliplatin
HR: Homologous recombination
HRD: Homologous recombination deficiency
HRR: Homologous recombination repair
HuR: Human antigen R
IRE: Irreversible electroporation
MMEJ: Microhomology mediated end joining
MMR: Mismatch repair
MTAP: Methylthioadenosine phosphorylase
NHEJ: Non-homologous end joining
OS: Overall survival
PAD: PARP+ATR+DNA-PK inhibitor regimen
PAD_tal_: Talazoparib-based PAD regimen
PALB2: Partner and localizer of BRCA2
PAR: Poly(ADP-ribose)
PARG: Poly(ADP-ribose) glycohydrolase
PARP: Poly(ADP-ribose) polymerase
PARPi: PARP inhibitor
PDAC: Pancreatic ductal adenocarcinoma
PDX: Patient-derived xenograft
POLQ: DNA polymerase theta
PRMT5: Protein arginine methyltransferase 5
PROTAC: Proteolysis-targeting chimera
RAD51: RAD51 recombinase
REV7: REV7 (also known as MAD2L2)
RPA: Replication protein A
STING: Stimulator of interferon genes
TNKS: Tankyrase
TP53: Tumor protein p53
WEE1: WEE1 G2 checkpoint kinase
